# Analysis of Hospitalization Cases for Filler Injection Complications: A Five‐Year Retrospective Study Among Iranian People

**DOI:** 10.1111/jocd.16733

**Published:** 2024-12-16

**Authors:** Ala Ehsani, Setareh Falakian, Amirhoushang Ehsani, Shahin Hamzelou, Kamran Balighi, Zahra Razavi, Zeinab Aryanian, Azadeh Khayyat, Parvaneh Hatami

**Affiliations:** ^1^ Medical Students, School of Medicine Shahid Beheshti University of Medical Sciences Tehran Iran; ^2^ Autoimmune Bullous Diseases Research Center Tehran University of Medical Sciences Tehran Iran; ^3^ Department of Dermatology, Razi Hospital Tehran University of Medical Sciences Tehran Iran; ^4^ Department of Dermatology Babol University of Medical Sciences Babol Iran; ^5^ PGY3 Resident Physician, Pathology Department of Medical College of Wisconsin Milwaukee Wisconsin USA

**Keywords:** complication management, cosmetic procedures, dermal fillers, hospitalization cases, hyaluronic acid

## Abstract

**Introduction:**

The skin is the largest organ system with many important clinical functions. Due to the increase in demand for cosmetic procedures and consequently the increase in complications from filler injections, this research aims to review the hospitalization cases involving filler complications.

**Methodology:**

This study retrospectively and cross‐sectionally reviews patient records hospitalized between the years 2016 to 2020, focusing on demographics (age, gender, residence), type of filler, type of complications, onset and duration of complications, duration of hospital stay, the person who administered the injection, and treatment outcomes. The goal is to understand the complications, prognosis, and potential risk factors.

**Results:**

A total of 58 patients were hospitalized due to complications arising from filler injections between 2016 and 2020. The average age of patients was approximately 37.79 years, ranging from 15 to 62 years old. A majority, 94.8%, were female and 62.1% were married. The most common filler used was hyaluronic acid, accounting for 81% of cases. Complications included cellulitis in 41.4% of cases, abscesses in 29.3%, granulomatous inflammation in 19%, and necrosis in 10.3%. The shortest hospital stay was 1 day and the longest was 9 days, with an average stay of approximately 2.33 days. In 65.5% of the cases, the injections were administered by medical professionals, and in 34.5%, by individuals without medical credentials. Married individuals generally sought hospital care later than others. Severe complications were more likely to occur when injections were administered by nonprofessionals. The time to seek medical attention was shorter for severe complications than for moderate ones. There were no deaths among the cases studied.

**Conclusion:**

The study highlights the variation in complication severity associated with filler injections performed by nonmedical personnel, emphasizing the importance of prohibiting such practices. Educating patients about the early signs of complications can significantly reduce severe outcomes and decrease antibiotic resistance.

## 1 Introduction

Not only is the skin by far the largest organ in the body, but it is also the chief protective barrier. The epidermis, dermis, and subcutaneous layer as three layers that make skin have properties and functions targeting the formation of protection against environmental aggression, maintenance of body temperature, and sensory sensitivity, which makes it one of the most complex, hence of importance in medical research in the field of dermatology and cosmetics. Demand for cosmetic enhancements has increased tremendously especially in noninvasive dermal fillers for the sake of appreciating their related complications [1].

Dermal fillers have gained great popularity because of their instant results and temporal nature. These characteristics of the process greatly attract customers who want to go through cosmetic rejuvenation but without actually going under the knife, Hyaluronic acid fillers are among the most commonly utilized ones due to the reason of its efficacy and relative safety [2]. But with benefits, increased use has undoubtedly raised the associated complications, which can extend anywhere from mild short‐term effects to disastrous ones on long‐term intensity.

This is reflected in the growing concern about such crimes traced in literature. The same study conducted by Trinh, McGuigan, and Gupta (2019) [3]; and Colon et al. 2023 reviewed the complications of sensitive area skin Injections into tear troughs and lips in a manner indicating the possibilities of both immediate and delayed side effects though along with Carella et al. (2022) give a clear view of the clinical interventions required for complications [2, 4].

The increase in dermal filler injection complications not only correlates directly with the rise in filler use, but also other factors such as changes in patient demographics, technique innovations, or improved reporting systems could be implicated as well. With growing clinical incidences of such complications and rising clinical significance, this article attempts to analyze 5‐year hospitalization cases of complications arising from filler injections. Through this analysis, we seek a better understanding of the frequency, nature, and severity in the hope that such understanding will serve not only to inform clinical practices but also future policy, as well as educational efforts geared toward the enhancement of patient safety involved in cosmetic procedures. This analysis is very important considering the increased use of dermal fillers and their associated risks are growing accordingly.

## 2 Method

In our retrospective cross‐sectional study, in 2016–2020, all clinical events due to filler injection complications were evaluated at a tertiary care hospital. The study's data‐gathering instrument was a structured prestudy survey. This checklist was prepared to comprise the whole record of data related to patient characteristics, including age, sex, and educational data; the clinical data such as the type of filler used, the site of injection, and the timing of the filler injection; the injector's educational data; the time frame, which include the period between injections and the beginning, including the complications and the time since the complication started and medical referral; and the clinical data such as duration of stay and whether surgery had been performed.

The following inclusion criteria were used: all data on the patients in the archives of the hospitals were selected based on their needs for authorizations between October 2022 and February 2023. The following exclusion criteria were applied during patient recruitment: patients without enough baseline information and incomplete records or missing data were excluded to avoid selection bias.

This study included More than 10 000 records that were checked to access, and the data were sent to SPSS software, version 26, for statistical analysis of descriptive statistics, frequency distribution‐related tables, and statistical charts describing the data. Means and standard deviations (SD) were calculated for continuous variables and frequency and percentage for categorical variables.

The Chi‐square test for categorical variables and the *t*‐test for quantitative variables were used for inferential statistics. A *p* value < 0.05 was set to demonstrate a meaningful difference and trends.

This thorough methodological approach aimed to ensure the reliability and validity of the findings, addressing potential data gaps through direct contact with patients when necessary. Ethical considerations were carefully maintained throughout the study, adhering to the Helsinki Declaration, with ethical approval granted under the identifier IR. TUMS.MEDICINE.1399.1224.

## 3 Result

This retrospective study evaluated 58 patients for complications from filler injections placed at a tertiary care hospital between 2015 and 2019. Of 58 patients, 55 patients, 94.8%, were women, and three patients, 5.2%, were men. The mean age of the patients was 37.79 ± 10.09 years, ranging from 15 to 62.

Among the patients, 22 persons corresponding to 37.9% were single, and 33 persons corresponding to 62.1% were married. Among the patients, 86.2% (50 cases) used temporary type fillers, 13.8% (8 cases) used permanent type fillers, 47 patients with hyaluronic acid fillers (94%), and three cases had collagen fillers. Of those with permanent fillers, four had fat injections, and four used silicone filler.

Among the 58 patients, physicians were the injectors at 65.5% of patients, followed by nonphysicians at 34.5%. Furthermore, there were 38 cases (65.5%) who were injected at a clinic while 20 cases (34.5%) were injected outside a clinic. The patient assessments in the period from injection to the onset of complications were as follows: immediate onset within 24 h after injection, early onset from 24 h to 4 months, and late‐onset after more than 4 months.

The minimum and maximum values concerning the number of days elapsed from injection to the onset of complications were 1 and 90 days, respectively, with an average of 14.8 days. About the sites of patients for which filler injection was performed, percent distribution and frequency for each site were as follows: The most common injection site was the head and face, with 54% and 93.1% of cases, respectively. Furthermore, three patients were hospitalized after the filler injection in the upper extremity, and one patient was hospitalized after the filler injection in the lower leg, primarily in the region of the gluteal muscle. No patient was hospitalized due to mild severity on account of severe complications. Most patients were hospitalized for moderately severe complications (62.1%).

In the study, 21 of 58 patients had a history of surgery (36.2%). Among the patients admitted with complications, 11 cases had granulation tissue, and 10 cases with abscesses (58.5% of these patients and 17.2% of all patients had undergone surgery). However, none of those with necrosis or cellulitis troubles had a history of surgery.

Age and surgery were not significantly associated. The number of hospitalization days was much fewer for surgical patients. Moreover, the days from injection to complication onset, complication onset to hospitalization, and the time from injection to hospitalization were much higher in the subjects who underwent surgery (Table 1).

**TABLE 1:** Analysis of the relationship between surgery and patient's age, number of hospitalization days, onset of complication, time from onset of complication to hospitalization, and time from injection to hospitalization.

Variable	Number of surgeries	Mean	Standard deviation	*p*
Age	No (37)	36.73	9.64	0.676
	Yes (21)	39.67	10.83	
Number of hospitalization days	No (37)	5.05	2.134	0.005
	Yes (21)	4.90	2.948	
Number of days from injection to onset of complication	No (37)	5.92	7.668	0.0005
	Yes (21)	30.29	28.720	
Number of days from onset of complication to hospitalization	No (37)	9.14	13.462	0.013
	Yes (21)	20.24	18.994	
Number of days from injection to hospitalization	No (37)	15.05	17.809	0.0005
	Yes (21)	50.52	42.011	

Among 41 cases of cellulitis, 90.2% (37 patients) were treated with a sulfamethoxazole‐trimethoprim combination, and 9.8% (4 patients) were treated with ciprofloxacin which was associated with higher rates of serious complications in the patients (*p* value = 0.05). Most of the sample was female (94.8%), and only 5.2% were male. No deaths were reported, most patients (93.1%) were discharged appropriately, while a few (6.9%) left the hospital without medical advice. Statistically significant differences were found between the onset of complications and hospital visits based on marital status, with single individuals seeking care earlier than married couples (*p* value = 0.008) and men admitted later than women when complications started later, which was statistically significant (*p* value = 0.05).

There is a significant correlation between permanent fillers and the time from the onset of complications to hospitalization (*p* value = 0.02), time from filler injection to the onset of complications (*p* value = 0.05), and time from injection to reaching the treatment, as all these intervals are long for permanent fillers (*p* value = 0.02).

In the patients evaluated in terms of onset of complications, most cellulitis and abscesses had immediate or early onset, necrosis had early onset, and granulation tissue had late onset (*p* value = 0.0001). Complications requiring surgery were more frequent in patients injected by nonclinicians (*p* value = 0.01). The study found an association between the severity of complications, number of days in hospital, and length of stay in cases with severe complications, but this association was not statistically significant.

In our study, there was a correlation between complication severity and the onset of complications, it was approximately 1.5 times greater in moderate complications, but the correlation was significant. Finally, in the study of the relationship between the severity of complications and the duration between injection and seeking care, this period was significantly shorter in severe complications (Table 2).

**TABLE 2:** The relationship between complication severity and the time between onset of complication and hospitalization, time between filler injection and the onset of complication, number of hospitalization days, and time between injection and hospital visit.

Variable	Severity	Number	Mean	Standard deviation	*p*
Time between onset of complication and hospitalization	Moderate	36	15.3	18.165	0.06
	Severe	22	10.9	12.92	
Time between filler injection and onset of complication	Moderate	36	17.64	24.64	0.006
	Severe	22	10	14.64	
Number of hospitalization days	Moderate	36	3.81	2.04	0.28
	Severe	22	6.95	1.64	
Time between injection and hospital visit	Moderate	36	32.67	36.48	0.005
	Severe	22	20.09	26.57	

The time between complication onset and hospitalization, time between filler injection and complication onset, length of time between injection and treatment seeking, and total number of hospitalization days were correlated with the ischemic nature of the lesions. Specifically, a higher number of hospital days in ischemic complications were detected, while the association between the number of days between injection and complication onset, the number of days between complication onset and hospitalization, and the number of days between injection and hospital are statistically insignificant (Table 3).

**TABLE 3:** Relationship between the ischemic nature of the complication and the following variables: The time between the onset of the complication and hospitalization, the time between filler injection and the onset of the complication, the time between injection and hospital visit, and the number of hospitalization days.

Variable	Number	Mean	Standard Deviation	*p*
Number of hospitalization days	Nonischemic: 52	4.69	2.36	0.002
	Ischemic: 6	7.67	1.033	
Time between filler injection and the onset of a complication	Nonischemic: 52	15.73	22.57	0.016
	Ischemic: 6	6.17	4.35	
Time between onset of complication and hospitalization	Nonischemic: 52	14.23	17.03	0.028
	Ischemic: 6	3.83	1.32	
Time between injection and hospital visit	Nonischemic: 52	29.96	34.68	0.003
	Ischemic: 6	10	3.89	

**FIGURE 1:**
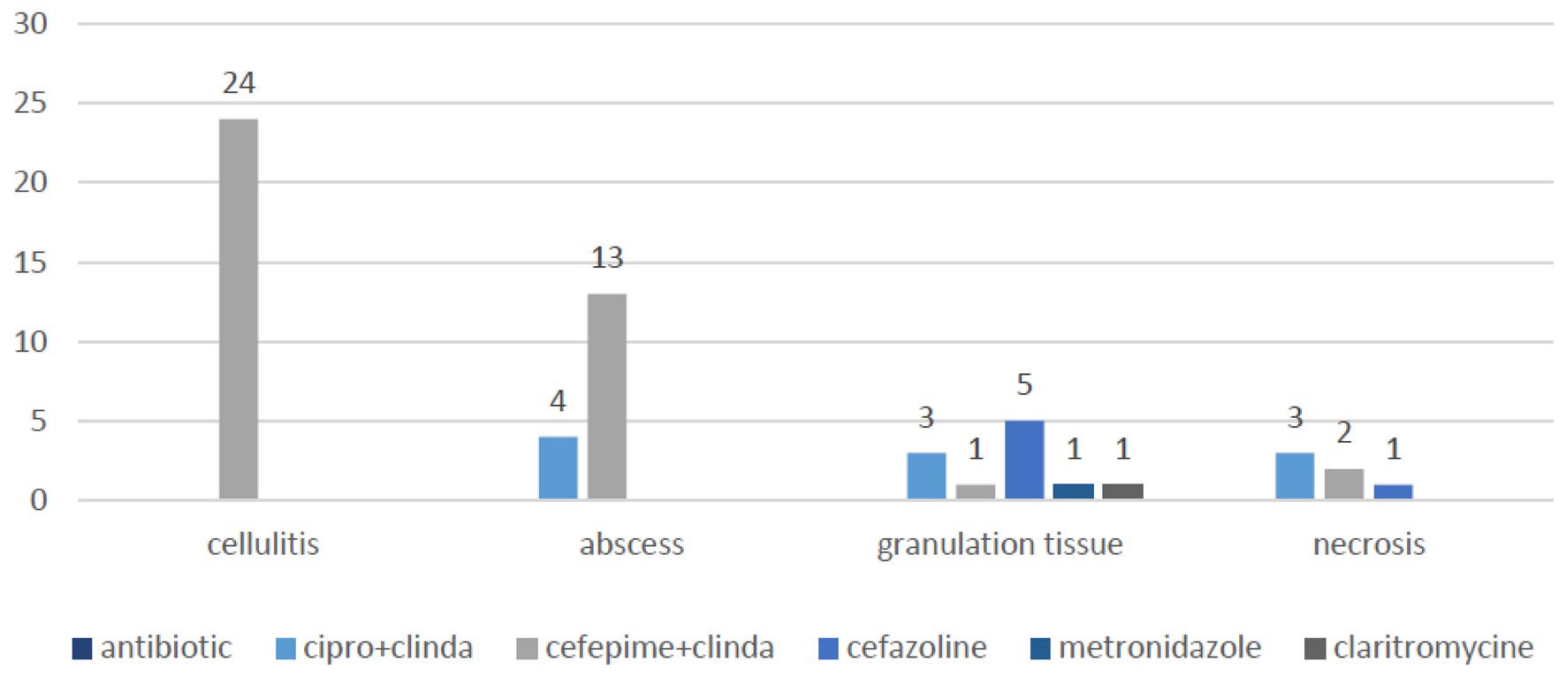
The frequency of each type of antibiotic based on the name of the complication.

Figure 1 shows the frequency of each antibiotic based on the type of complication. The combination of cefepime and clindamycin is common in the treatment of cellulitis and abscesses, but rare in other types of complications (*p* value = 0.0001).

The incidence of complications such as necrosis, granulation tissue, and abscesses was significantly higher in nonclinician injectors (*p* value = 0.02).

Twenty‐three of 58 patients were treated with hyaluronidase, equivalent to 39.6% of all patients, and 48.9% of hospitalizations due to hyaluronic acid injections. In 47 cases with hyaluronic acid filler, there was a significant association between hyalase administration and the severity of complications, such that hyalase was more frequently prescribed to patients (*p* value = 0.05).

**FIGURE 2:**
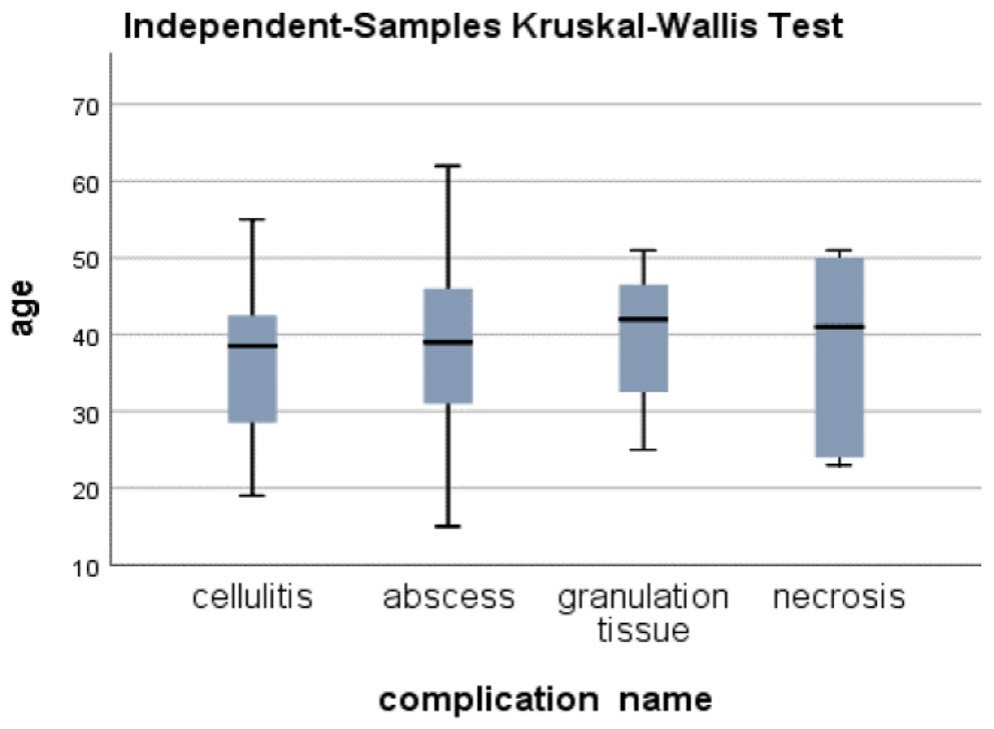
The relationship between age and the condition's name: Cellulitis, abscess, granulation tissue, necrosis.

There were no significant correlations between patient age and the type of filler used (*p* value = 0.73), the onset timing of complications (*p* value = 0.28), the severity of complications (*p* value = 0.78), specific complications developed such as cellulitis, abscess, granulomatous inflammation, or necrosis (*p* value = 0.74) (Figure 2), or the time from complication onset to hospital visit (*p* value = 0.29).

The association between patient age and surgery was not significant (*p* value = 0.67), nor did patient age and hyalase use show any association with a *p* value of insignificance level of 0.56 between the two.

No statistically significant differences were also observed in the age and the filler injection, due to increased age, with a *p* value of 0.62.

There was no significant association between the severity of the complications and the marital status of the patients, with *p* values of 0.29 and 0.71, respectively.

No significant differences were also found in complications based on provider education, as presented in the *p* value of 0.56. Similarly, no significant association was found between the patient's sex and providers' type, with a *p* value of 0.26.

The average length of hospital stay was 5.27 days for patients from Tehran (The capital city) and 4.25 days for others. However, no significant association was found between length of hospital stay and place of residence, with a *p* value of 0.96.

Marital status showed no significant association with time from symptom onset to admission, time from filler injection to complication resolution, total hospital stays (*p* value = 0.68), onset of symptoms to admission (*p* value = 0.26), or total hospital stay (*p* value = 0.68).

The relationship between temporary filler and the time of complication emergence showed a *p* value of 0.077 compared to permanent fillers; complications arose earlier. On average, the complication happened earlier in temporary fillers, and it happened after a longer time in permanent fillers.

No significant association of fillers such as hyaluronic acid, silicone, and others, with the onset of complications was found, as the *p* value was 0.16.

There was no significant association between temporary fillers and the type of ischemia and other complications (*p* value = 0.14). There was no significant association between the type of ischemia and other complications and the temporary filler (*p* value = 0.49), time of complication onset (*p* value = 0.28), site of filler injection (*p* value = 0.78), the filler injector type (*p* value = 0.17), or patient age (*p* value = 0.21).

There was no relationship between the site of injection and complication severity (*p* value = 0.71), even with the type of complication (*p* value = 0.81), and there was no association at all with injection type, no matter how sensitively it is seen (*p* value = 0.96).

No strong correlation was indeed significant for the intensity of complications and the following factors: filler type with a *p* value of =0.90; age, with a *p* value of 0.14; sex, with a *p* value of 0.29; site of filler injection, with a *p* value = 0.71; surgery, with a *p* value of 0.25; and type of antibiotics, with a *p* value of 0.30, aspirin with a *p* value of 0.77, nitroglycerin with a *p* value of 0.29, prednisolone with a *p* value of 0.76, and acyclovir with a *p* value = 0.12.

An association was found between hyalase utilization and complication severity (*p* value = 0.07). The complication type and outcome of the admission were not significantly related (*p* value = 0.39).

The age and the complication type were also not significantly related. Hospital days were significantly more for necrosis and abscesses compared with cellulitis and granulation tissue.

The mean days from injection to the onset of complications, the mean days from the onset of the complication to hospital admission, and the mean days from injection to discharge after being admitted to the hospital were significantly higher in granulation cases than in others.

There was no significant association between undergoing surgery and the provider type (*p* value = 0.16). Among the 17 abscess cases, there was no significant difference in the number of days in the hospital based on whether surgery was performed or not (*p* value = 0.23).

Our findings underscore the importance of monitoring patient outcomes and adherence to best practices in filler injections to mitigate risks and enhance patient safety.

## 4 Discussion

Analysis of clinical data from filler injection complications over 4 years provides important insights into the nature, function, and outcome of these adverse events This study involved 58 patients, predominantly female (94.8%). This gender difference may stem from the heightened sociocultural pressures on women to attain physical appeal and adhere to societal standards of beauty. This phenomenon is particularly relevant in the context of Iran, where distinct gender‐specific social and cultural norms prevail.

Our finding that temporary fillings, especially hyaluronic acid, are associated with the majority of complications (86.2%) is consistent with previous reports. A study by Rohrich et al. (2011) also identified hyaluronic acid filler as the most common cause of complications due to its widespread use in aesthetic practices [5].

Despite its popularity, our results show a high risk of complications, including cellulitis, abscess formation, and necrosis, which is consistent with the findings of Kim, Sykes, and Hyun (2014) [6]. However, it should be noted that although some studies, such as that of Sundaram et al., (2015), reported a very low incidence of serious complications with hyaluronic acid fillers, our study shows that if complications do occur, they may be severe enough to require hospitalization [7].

A notable finding was that patients who received silicone filler injections and other routine fillers had a significantly longer time between the onset of complications and subsequent hospitalization in our study. The mean time from injection to the onset of discomfort was 14.8 days, but this was significantly longer for regular injections (*p* = 0.05). This delay is consistent with the observations of Signorini and Liu (2016), who reported that permanent fillers have late‐onset complications, often with more severe outcomes due to the consistency of these factors with regular fillers characteristics by Cassuto and Ancona (2016) highlighted above, which can lead to chronic inflammation and granuloma formation even years after injection [8, 9]. Our findings underscore the importance of careful patient selection and comprehensive counseling when considering dosing, emphasizing the importance of regularity.

Another important aspect is the significant association between injector type and the severity of complications observed in our study. Unfortunately, injections by nonmedical personnel such as those who work in hair salons under a nonmedical setting which is an illegal and unethical process have been increasingly reported in some areas due to lower socioeconomic status of patients. Nonphysician injections were more likely to cause serious complications, including abscesses and necrosis (*p* = 0.02). This is consistent with the findings of De Boulle and Heydenrych (2015), who highlighted an increased risk of complications when individuals with inadequate medical training administered dermal fillers, and the study of Funt and Pavicic (2013) focuses on the role of injector education in reducing the risk of adverse events, particularly vascular complications which are often linked to improper injection techniques, particularly with injection procedures of the oral anatomy, may be due to insufficient knowledge of oral anatomy, inappropriate technique, or inadequate posttreatment care, again highlighting the need for regulation and education in this rapidly developing field [10, 11].

Consistent with our study, several reports have emphasized the importance of timely intervention in managing complications related to filler‐related. For example, the use of hyaluronidase to treat complications with hyaluronic acid fillers was common in our study, with 39.6% of patients receiving this treatment.

The significant correlation between hyaluronidase use and the severity of complications indicates that although hyaluronidase is effective in managing adverse events, its importance tends to indicate more severe complications The findings comparison reported by Cohen et al. (2013), who also noted that the need for hyaluronidase often indicates serious complications that require careful management. Furthermore, a study by King, Pezeshk, and Delaney (2015) highlighted the importance of early application of hyaluronidase to reduce vascular inhibitory effects, further supporting our findings [12, 13].

Interestingly, our study showed no significant association between patient age and the nature or severity of problems, which is consistent with some reports suggesting that older patients may be at increased risk for access adverse outcomes due to age‐related changes in skin tissue structure, for example, Monheit, Coleman, and Rohrich (2016) suggested that older patients may be at increased risk due to decreased elasticity of tissues and presence of pre‐existing conditions but other studies, such as Swift and Remington (2017) suggest that the proper technique and filler choice, age does not significantly impact complication rates, which may explain the lack of association found in our study. The small sample size and specific demographic characteristics of our study population may account for this discrepancy [14, 15].

Our findings regarding ischemic complications were particularly concerning. Ischemic complications, though less common, were associated with a significantly higher number of hospitalization days and shorter intervals between complication onset and hospitalization (*p* = 0.002). This finding underscores the severity of vascular complications following filler injections and echoes the concerns raised by Beleznay et al. (2015), who emphasized the importance of prompt recognition and treatment of vascular complications to minimize adverse outcomes. Furthermore, our study's results align with a review by Pavicic et al. (2014), which highlighted the critical nature of early intervention in cases of vascular occlusion to prevent tissue necrosis and other severe outcomes [16, 17].

Another area of comparison is the prevalence of complications related to filler type. While our study found that hyaluronic acid fillers were most commonly associated with complications, with a significant portion of patients requiring hospitalization, other studies, such as the one by Micheels et al. (2012), have reported a lower overall complication rate for hyaluronic acid fillers. However, Micheels et al. (2012) also acknowledged that when complications do occur, they can be severe, particularly in cases of vascular occlusion or infection, which our study supports [18].

The role of patient education and informed consent cannot be overstated. Our findings suggest that many patients were unaware of the potential risks associated with filler injections, particularly when performed by nonclinicians. This is consistent with the observations by Trévidic and Pönyai (2017), who found that comprehensive patient education significantly reduces the risk of complications by ensuring patients understand the importance of choosing a qualified injector and recognizing early signs of complications [19].

Despite the success of this study, some limitations must be considered, including small sample size, absence of a control group, and the retrospective cross‐sectional design of the study, which inherently limits the ability to infer causality, as well as inevitable occurrence of recall bias and the inability to control for confounding variables such as patient health status, socioeconomic factors, and injector experience.

Hence, future prospective studies or controlled trials are needed to address the limitations of the retrospective design and explore complications outside of hospitalization contexts.

In conclusion, this study adds to the growing body of evidence on filler injection complications, highlighting the need for vigilance in both the administration and management of these procedures. Our results are consistent with findings from various studies, emphasizing the importance of qualified injectors, addressing regulatory deficiencies, mandatory injector certification, updated injector training courses, patient education, and adherence to clinical guidelines to minimize the risk of severe complications.

We promptly suggest specific patient education programs or campaigns to inform the public about the risks of filler injections and the importance of seeking qualified injectors.

Further research is warranted to explore the long‐term outcomes of patients experiencing filler complications and to develop strategies for preventing these adverse events.

## 5 Clinical Implications

The findings underscore the importance of standardized injection practices, provider qualifications, and vigilant postprocedural monitoring to mitigate the risk of severe complications associated with filler injections. Future research should focus on prospective studies to validate these findings and refine guidelines for safer aesthetic procedures.

By integrating these insights with existing literature, clinicians can better tailor patient management strategies and enhance outcomes in aesthetic medicine.

## Author Contributions

A.E., Z.R., A.E., and S.F. performed the research. S.H., P.H., and K.B. designed the research study. Z.A. and A.E. supervised the project. A.E., Z.R., and S.H. analyzed the data. P.H. and A.K. wrote the paper.

## Conflicts of Interest

The authors declare no conflicts of interest.

## Data Availability

The data that support the findings of this study are available from the corresponding author upon reasonable request.
